# A *DRD2/ANNK1*–*COMT* Interaction, Consisting of Functional Variants, Confers Risk of Post-traumatic Stress Disorder in Traumatized Chinese

**DOI:** 10.3389/fpsyt.2018.00170

**Published:** 2018-04-30

**Authors:** Kunlin Zhang, Li Wang, Chengqi Cao, Gen Li, Ruojiao Fang, Ping Liu, Shu Luo, Xiangyang Zhang, Israel Liberzon

**Affiliations:** ^1^Laboratory for Traumatic Stress Studies and Center for Genetics and BioMedical Informatics Research, CAS Key Laboratory of Mental Health, Institute of Psychology, Beijing, China; ^2^Department of Psychology, University of Chinese Academy of Sciences, Beijing, China; ^3^Shenzhen Key Laboratory of Affective and Social Cognitive Science, Shenzhen University, Shenzhen, China; ^4^People's Hospital of Deyang, Deyang, China; ^5^Department of Psychiatry, University of Michigan and Mental Health Service, VA Ann Arbor Healthcare System, Ann Arbor, MI, United States; ^6^Department of Psychology, University of Michigan, Ann Arbor, MI, United States

**Keywords:** post-traumatic stress disorder (PTSD), dopaminergic genes, *DRD2/ANNK1*–*COMT* interaction, modulate, functional variants

## Abstract

**Objective:** Post-traumatic stress disorder (PTSD) is a trauma- and stress-related psychiatric syndrome that occurs after exposure to extraordinary stressors. The neurotransmitter dopamine (DA) plays important roles in neurobiological processes like reward and stress, and a link between PTSD and the dopaminergic system has been reported. Thus, the investigation of an association between PTSD and gene–gene interaction (epistasis) within dopaminergic genes could uncover the genetic basis of dopamine-related PTSD symptomatology and contribute to precision medicine.

**Methods:** We genotyped seven single nucleotide polymorphisms (SNPs) of three dopaminergic genes *DRD2/ANNK1* (rs1800497 and rs1801028), *COMT* (rs6269, rs4633, rs4818 and rs4680) and *DBH* (rs1611115), in a Chinese predominantly adult cohort that had been exposed to an earthquake (156 PTSD cases and 978 controls).

**Results:** Statistical genetics analysis identified a *DRD2/ANNK1*–*COMT* interaction (rs1800497 × rs6269), which is associated with PTSD diagnosis (*P*_interaction_ = 0.0008055 and *P*_corrected_ = 0.0169155). Single-variant and haplotype-based subset analyses showed that rs1800497 modulates the association directions of both the rs6269 G allele and the rs6269-rs4633-rs4818-rs4680 haplotype G-C-G-G. The interaction (rs1800497 × rs6269) was replicated in a Chinese young female cohort (32 cases and 581 controls, *P*_interaction_ = 0.01329).

**Conclusions:** Rs1800497 is related to the DA receptor D2 density and rs6269-rs4633-rs4818-rs4680 haplotypes affect the catechol O-methyltransferase level and enzyme activity. Thus, the interaction was inferred to be at protein–protein and DA activity level. The genotype combinations of the two SNPs indicate a potential origin of DA homeostasis abnormalities in PTSD development.

## Introduction

According to the fifth edition of *Diagnostic and Statistical Manual of Mental Disorders* (DSM-5), post-traumatic stress disorder (PTSD) is diagnosed if a patient presents with at least one out of five intrusive symptoms, one out of two active avoidance symptoms, two out of seven negative alterations in cognitions and mood and two out of six alterations in arousal and reactivity symptoms; each must persist for at least 1 month after trauma exposure (1). The World Mental Health Surveys reports that the lifetime prevalence of PTSD is 3.9% in the general population and 5.6% among trauma-exposed individuals (2). Previous studies suggested that genetic factors play an important role in the development of PTSD (3–5). The heritability of PTSD is estimated to be 40% by a twin study (4) and 29% (specific for European-American females) by a molecular genetic study (6). Numerous smaller studies of genetic variants, mostly single nucleotide polymorphisms (SNPs), of PTSD candidate genes have been carried out, examining main genetic or gene–environment interaction effects of candidate genes on PTSD. Through these studies, more than 50 genes have been explored to investigate the association between them/their variants and PTSD; for each of these at least one significant result has been obtained. Among them, the most frequently studied genes are *SLC6A4, SLC6A3, ADCYAP1R1*, dopamine receptor D2 (*DRD2*), *FKBP5, COMT* and *BDNF*, which were represented in more than 15 studies. Further, the 5-HTTLPR variant of *SLC6A4* was explored in a total of 41 studies and in 13 of them significant association test results were obtained. The variant rs6265 of *BDNF* was investigated by 10 studies and three of them showed significant results. However, the findings had not been uniformly replicated (7). Furthermore, genome-wide association studies (GWASs) using large sample sizes have been carried out in search of candidate SNPs conveying a higher PTSD risk (6–8). Particularly, the most recent GWAS of PTSD by the Psychiatric Genomics Consortium (PGC) combined 11 multiethnic studies to form a large-scale analysis (6). However, the overall number of PTSD genetic studies is small and the number of reported PTSD susceptibility genes is limited in comparison to some other mental diseases (7, 9), like schizophrenia, major depression disorder (MDD) and bipolar disorder (BD) (10–12). *N* values are typically relatively small, underscoring the need for further research of the genetic mechanisms behind PTSD.

From the point view of precision medicine, the concept is to prevent, diagnose and treat disease by analyzing individual genomic data and some other individual biomedical information. The identification and detection of the risk-enhancing SNPs furthers the cause of precision medicine, in particular aiding the early prevision of the disease (13). Most published genetic studies of PTSD to date are candidate gene association studies which focus on the relationship between PTSD diagnoses/symptoms and single gene loci. However, from the point view of system biology, the interaction of genes is as important for understanding the resulting phenotype as single gene effects. Single candidate gene association studies often miss important information like gene–gene interactions (epistasis) (14–16); such interactions may contribute to a biological basis of PTSD development (3, 17). It can be particularly difficult to identify these interaction effects using traditional statistic tests for multiple comparisons in GWAS-style studies. Epistasis screening in a hypothesis-driven manner is a solution to this problem (16), and we propose to do as such in the study of PTSD.

Dopamine (DA) is a neurotransmitter that plays an important role in neurological processes such as reward, motivation and stress (18–20). Dysregulation of dopaminergic function and signaling has been associated with a number of psychiatric disorders like schizophrenia, drug and alcohol dependence (21), attention deficit hyperactivity disorder (ADHD) (22) and possibly PTSD (23). Genes regulating DA neurotransmission, such as the catechol-O-methyltransferase (*COMT*) gene, the *DRD2* gene and the DA beta-hydroxylase (*DBH*) gene, are involved in biological processes of DA signaling (degradation, transport, signal transduction, etc.) and must maintain DA homeostasis through their interaction with each other. These genes have been “hot” candidate genes for susceptibility of being involved in mental disorders (24) including PTSD (7). The *DRD2* gene encodes DRD2, which inhibits adenylate cyclase; some important genetic variants, such as TaqIA (rs1800497) altering expression of both *DRD2* and its neighborhood gene *ANNK1*, and consequently the *DRD2*/*ANNK1* locus constitutes a plausible target for genetic studies. The *COMT* gene encodes COMT, which catalyzes the transfer of a methyl group, from S-adenosylmethionine to catecholamines (causing DA inactivation). DBH is encoded by the *DBH* gene and it converts DA to norepinephrine (DA conversion). Although some work has been done on the relationship between PTSD and these genes, the results are inconsistent and difficult to replicate (25). One possible explanation for the mixed results can be unaddressed gene–gene interaction effects between the dopaminergic genes. We believe the investigation of the association between PTSD and gene–gene interactions of dopaminergic genes could be a feasible way to examine contribution of the DA system to PTSD development.

In the present study, we have investigated gene–gene interactions of the three dopaminergic genes, *DRD2/ANNK1, COMT* and *DBH*. We genotyped seven SNPs of the three loci in a Chinese predominantly adult cohort that had been exposed to the 2008 Whenchuan earthquake and identified *DRD2*/*ANNK1*–*COMT* interaction that was associated with PTSD. Combining with the available experimental evidence, we inferred the gene–gene interaction from SNP–SNP level to protein–protein level and DA homeostasis level.

## Materials and methods

### Sample and procedure

The samples of the present study were recruited from a large rebuilt community in Hanwang Town, Mianzhu City, China. Hanwang Town was almost completely destroyed by the 2008 Wenchuan earthquake; we conducted the survey five and a half years after the earthquake. Details of the cohort have been described somewhere else (26, 27). Participates were predominantly adult Chinese (≥16 years old) who personally experienced the deadly earthquake. Individuals with mental retardation or a major psychiatry history (like organic mental disorders and schizophrenia) were excluded from our study. PTSD symptoms were measured using the PTSD Checklist for DSM-5 (PCL-5), which is a 20-item well validated self-reported scale to capture the *DSM-5* PTSD symptoms with excellent reliability (28, 29). According to the DSM-5 diagnostic criteria, the presence of at least one intrusion symptom, one avoidance symptom, two negative alterations in cognitions and mood symptoms and two arousal symptoms endorsed as 2 or greater was used to identify possible PTSD cases. Earthquake-related trauma exposure was measured by a ten-question scale (26). Each item was rated either 0 (not experienced) or 1 (experienced) and the sum score was calculated as the level of trauma exposure. After participants completed the self-report questionnaires, peripheral blood samples were collected and DNA was extracted for genotyping. A total of 1,140 subjects with both clinical and genotyping data were initially included. We further excluded six subjects due to missing sex information, resulting in a total of 1,134 subjects—156 PTSD cases and 978 controls. More than 99.6% of our study subjects were Han Chinese. We also recruited a separate replication cohort consisting of female middle school students ranging from 13–17 years old that had experienced the 2008 Wenchuan earthquake (32 PTSD cases and 581 controls). Their PTSD diagnosis was also inferred from PCL-5 and their DNA was extracted from the saliva. This cohort was utilized for testing the replication of epistasis (i.e., rs1800497 × rs6269). Our study was approved by the Institutional Review Board of Institute of Psychology, Chinese Academy of Sciences. For the predominantly adult Chinese cohort, informed consents were signed by all participants. Among them, two were younger than 18 years old and they signed informed consents with permission of their guardians. For the replication cohort, informed consents were signed by all participants' guardians, with agreements of all participants. The demography of all the subjects is listed in Table 1.

**Table 1:** Demography of subjects in our study.

**Data set**	**No. of samples (female/male)**	**Age of samples (mean ± sd)**	**Range of age**
**DISCOVERY COHORT**			
Case	156 (115/41)	52.53 ± 8.59	21–66
Control	978 (658/320)	47.37 ± 9.98	16–73
Total	1134 (773/361)	48.08 ± 9.96	16–73
**REPLICATION COHORT**			
Case	32 (32/0)	14.22 ± 0.71	13–15
Control	581 (581/0)	14.28 ± 0.83	13–17
Total	613 (613/0)	14.27 ± 0.82	13–17

### Variants selection, genotyping, and quality control

We chose three important dopaminergic gene loci and their corresponding important functional SNPs, *DRD2/ANNK1* (rs1800497 and rs1801028), *COMT* (rs6269, rs4633, rs4818, and rs4680), and *DBH* (rs1611115). The detailed information, including chromosome position and possible function, of the SNPs is listed in Supplementary Table 1. The genotyping was conducted by a custom-by-design 2 × 48-Plex SNPscan^TM^ Kit (Genesky Biotechnologies Inc., Shanghai, China) based on a double ligation and a multiplex fluorescence PCR. A Hardy-Weinberg equilibrium (HWE) test was performed by exact tests of the HWE with efficient computational methods (30) implemented in PLINK (31). The results of the HWE test are shown in Supplementary Table 1. For our quality control (QC) of the samples, we removed subjects with call rate <0.8. QC of SNPs was performed by excluding SNPs with call rate <0.95 or minor allele frequency (MAF) < 0.01 in either cases or controls or an HWE test *P* < 0.05 for controls. All the subjects and SNPs passed this standard.

### Gene–gene interaction screening and follow-up logistic regression analysis

All of our analyses were performed by PLINK v1.07 and 1.09 (v1.07 was only for haplotype-based analysis) (31), Haploview v4.2 (32), and R3.2.3 (https://www.r-project.org/). All the logistic regression models utilized an additive model (count of the minor allele for each SNP) and *P*-values of the *t*-test for logistic regression were two-tailed. We first performed a single SNP-based analysis using a logistic regression model including SNP, gender, age and trauma exposure. The interaction between SNP and trauma exposure was further included in the regression model. Then we carried out epistasis analysis to screen the 21 pairwise interactions formed by the seven SNPs with a simple logistic regression model, using the following formula:

$$\begin{array}{l} \text{log}\left(\text{p}/\left(1-\text{p}\right)\right)\;=\text{ SNP}1+\text{SNP}2+\text{SNP}1\times \text{SNP}2. \end{array}$$

The interaction *P*-value (*P*_interaction_) was calculated and subjected to Bonferroni correction for multiple comparisons. Afterwards, for the SNP pairs with *P*_corrected_ < 0.05, we applied an initial logistic regression model:

$$\begin{array}{l} \text{log}\left(\text{p}/\left(1-\text{p}\right)\right)=\text{gender}+\text{age}+\text{SNP}1+\text{SNP}2+\text{SNP}1\\ \times \text{ SNP}2+\text{trauma exposure}+\text{SNP}1\\ \times \text{ trauma exposure}+\text{SNP}2\\ \times \text{ trauma exposure}+\text{SNP}1\\ \times \text{ SNP}2\;\times \text{ trauma exposure}. \end{array}$$

A following Akaike Information Criterion (AIC)-based model selection procedure was performed using a stepwise algorithm (33, 34) implemented in R to obtain the optimized logistic regression model. In our study, the final optimized model was

$$\begin{array}{l} \text{log}\left(\text{p}/\left(1-\text{p}\right)\right)=\text{gender}+\text{age}+\text{SNP}1+\text{SNP}2+\text{SNP}1\\ \times \text{ SNP}2+\text{trauma exposure}. \end{array}$$

The final model was applied to all the following logistic regression analyses. In addition, we further analyzed the gene–gene interactions in females and males respectively considering the important role of gender in PTSD genetic studies.

### Subset analysis

The gene–gene interaction screening revealed an interaction between rs1800597 and rs6269, thus we performed a secondary analysis to further understand how these SNPs interact. We divided the full data set into three subsets based on a single SNP genotype, then tested the association of the other SNP in the three subsets. When we tried to split the full data set by the rs6269 genotypes GG, GA and AA, we found that rs1800497 was only associated with PTSD (*P* < 0.05) in the rs6269 GG set, which had a relatively small sample size (19 cases and 100 controls). Thus we decided to divide the full data set according to the genotype of rs1800597 (AA, AG, and GG).

We firstly utilized a chi-squared test for the contingency table to examine the difference of gender distribution among subsets and Analysis of Variance (ANOVA) to examine the age difference among subsets. Then we performed chi-square tests (genotypic and allelic model, respectively) and logistic regression for rs6269 in each subset and the full set. The logistic regression model followed the abovementioned optimized model. For all the logistic regressions with additive model of minor allele count per person, we coded AA/AG/GG as 2/1/0 for rs1800497 and GG/GA/AA as 2/1/0 for rs6269 respectively. Besides the SNP analysis, we further performed haplotype-based analysis for haplotypes of rs6269-rs4633-rs4818-rs4680 for each subset and the full set. A linkage disequilibrium (LD) plot was drawn in Haploview. We then estimated the haplotype frequency by an expectation maximization (EM) algorithm implemented in PLINK and performed logistic regression with gender, age and trauma exposure as covariates to examine the haplotype-PTSD association. To guard against bias due to the relatively small sample size, we employed a permutation analysis with 1,000,000 permutation cycles to verify the results of the subset analyses.

## Results

### Gene–gene interaction screening and follow-up logistic regression analysis

The genotype frequency (GF) of the seven SNPs in cases and controls is shown in Supplementary Table 1. For single SNP-based analysis, none of the SNPs showed either main effect or gene–environment interaction effects for PTSD (all *P* > 0.05, see Supplementary Table 2). The results of the 21 pairwise interaction tests are shown in Supplementary Table 3. Two gene–gene interactions, rs1800497 × rs6269 (*P*_interaction_ = 0.0008055 and *P*_corrected_ = 0.0169155) and rs1800497 × rs4818 (*P*_interaction_ = 0.001008 and *P*_corrected_ = 0.021168), met the corrected significance threshold of *P*_corrected_ < 0.05. Since rs6269 was in linkage disequilibrium (LD) with rs4818 (*r*^2^ = 0.94, calculated by Haploview and based on our cohort), the two interactions indexed the same signal and we only focused on the most significant result (rs1800497 × rs6269, which denoted a *DRD2*/*ANNK1*–*COMT* interaction) in following analyses. The results of the initial model for this SNP pair are listed in Supplementary Table 4. After model optimization, only gender, age, trauma exposure, rs1800497, rs6269 and rs1800497 × rs6269 remained in the final optimized model (Table 2). The AIC of the optimized model was 840.0, less than the AIC of the initial model (844.3). In the optimized model, the interaction between rs1800497 and rs6269 remained significant (*P* = 0.00201). The analysis results for females and males are shown in Tables 3, 4, respectively. For female subjects, the results were quite similar to those of the overall subjects (*P*_interaction_ = 0.00133), while for males, the result in total sample was not replicated (*P*_interaction_ = 0.23171). These results suggest that this gene–gene interaction might be gender-specific and only exist in females.

**Table 2:** Summary of the optimized final logistic regression model of rs1800497 × rs6269.

**Variable**	**beta**	**Std. Error**	***t*-value**	***P*-value**
rs1800497 × rs6269	0.60563	0.19606	3.089	0.00201
rs1800497	−0.23301	0.18536	−1.257	0.20872
rs6269	−0.54082	0.22672	−2.385	0.01706
Gender	0.53005	0.20212	2.622	0.00873
Age	0.07203	0.01076	6.692	2.20*e*−11
Trauma exposure	0.25429	0.04931	5.157	2.51*e*−07

*For the logistic regressions with additive model of minor allele count per person, we coded AA/AG/GG as 2/1/0 for rs1800497 and GG/GA/AA as 2/1/0 for rs6269 respectively. This applies to all the following tables*.

**Table 3:** Logistic regression analysis of rs1800497 × rs6269 for female subjects.

**Variable**	**beta**	**Std. Error**	***t*-value**	***P*-value**
rs1800497 × rs6269	0.74833	0.23311	3.210	0.00133
rs1800497	−0.54665	0.22980	−2.379	0.01737
rs6269	−0.58393	0.26002	−2.246	0.02472
Age	0.08408	0.01270	6.622	3.55*e*−11
Trauma exposure	0.23222	0.05776	4.021	5.80*e*−05

**Table 4:** Logistic regression analysis of rs1800497 × rs6269 for male subjects.

**Variable**	**beta**	**Std. Error**	***t*-value**	***P*-value**
rs1800497 × rs6269	0.46424	0.38817	1.196	0.23171
rs1800497	0.32976	0.33296	0.990	0.32198
rs6269	−0.64157	0.49243	−1.303	0.19262
Age	0.04506	0.02182	2.065	0.03895
Trauma exposure	0.28493	0.09813	2.904	0.00369

### Subset analysis

**Figure 1:**
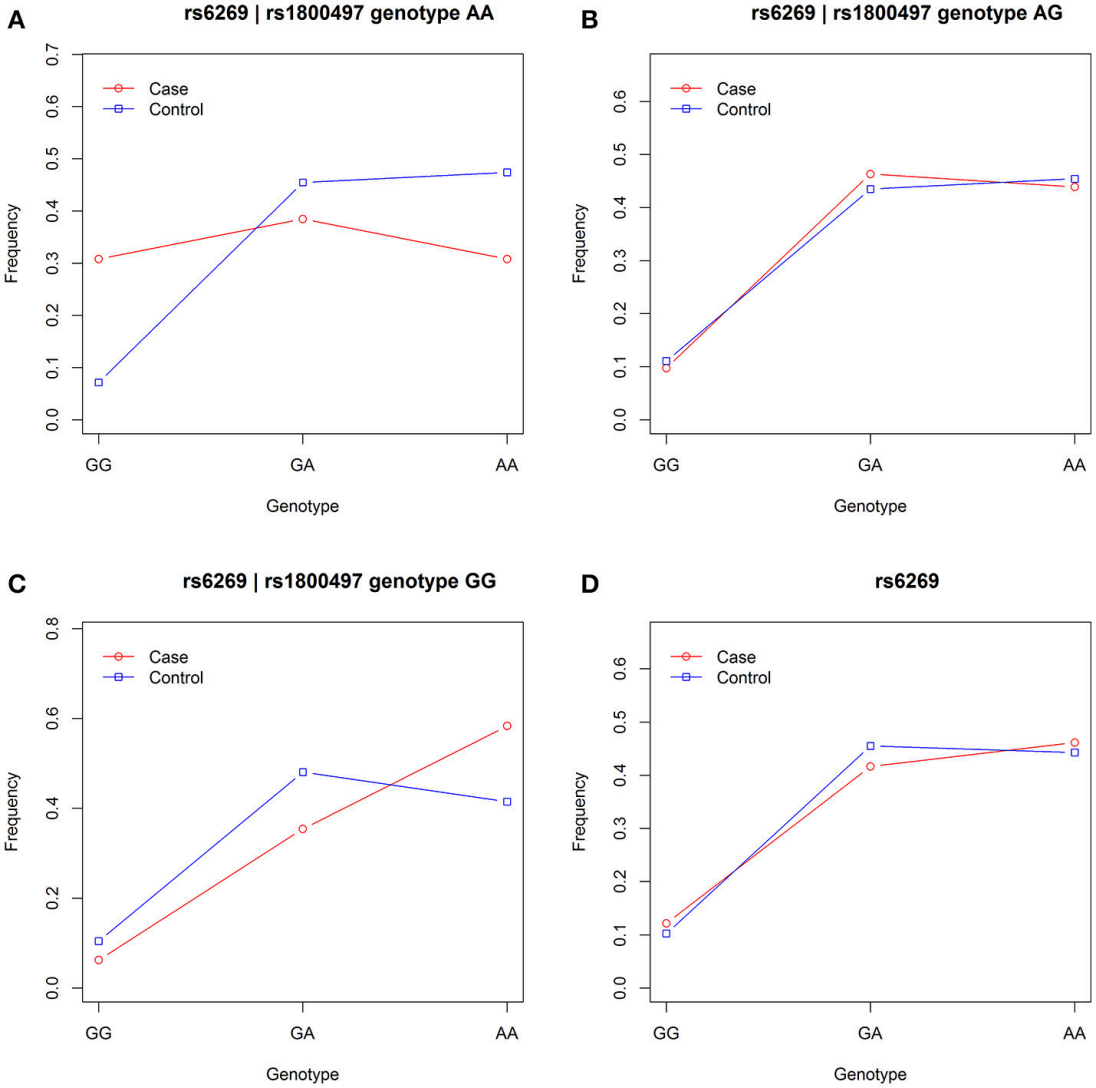
Genotype frequency variability of rs6269 between case and control in the rs1800497 AA set, the rs1800497 AG set, the rs1800497 GG set and the full set **(A–D)**, respectively.

We grouped our sample into three subsets, the rs1800497 AA set (individuals with rs1800497 genotype AA), the rs1800497 AG set (individuals with rs1800497 genotype AG) and the rs1800497 GG set (individuals with rs1800497 genotype GG). The demography (gender and age) of the three subsets and full set are in Supplementary Table 5. There was neither a gender distribution difference among subsets nor an age difference among subsets (χ^2^ = 0.004392, *df* = 2 and *P* = 0.9978; *F* = 0.75, *df* = 2 and *P* = 0.47, respectively). Figure 1 shows the genotype frequency variability of rs6269 between case and control for the four sets. There was obvious rs6269 genotype frequency variability in the rs1800497 AA set and GG set, with different patterns. The genotype frequency variability of the rs1800497 AG set and the full set was negligible. The chi-squared test results for rs6269 in the three subsets and the full set are shown in Table 5. The rs6269 allele was found to be differentially distributed between PTSD cases and controls in the rs1800497 AA (*P*_allelic_ = 0.004232) and rs1800497 GG sets (*P*_allelic_ = 0.03974), which implies a gene–gene interaction effect. Logistic regression analysis (see Table 6) further indicated that rs1800497 modulates the association directions between the rs6269 genotype and PTSD (rs1800497 AA set: rs6269 minor allele G associated with increased PTSD risk, OR = 2.32 and *P* = 0.010398; rs1800497 AG set: not significant; rs1800497 GG set: rs6269 minor allele G associated with decreased PTSD risk, OR = 0.56 and *P* = 0.03201). The result in the total sample was only replicated with the female sample (see Supplementary Table 6) but not with the male sample (see Supplementary Table 7). All of the aforementioned results were confirmed by permutation tests (Tables 5, 6 and Supplementary Tables 6, 7).

**Table 5:** Chi-squared test of rs6269 in the rs1800497 AA set, the rs1800497 AG set, the rs1800497 GG set and the full set, respectively.

**rs1800497 genotype**	**rs6269 genotype count (GG/GA/AA) Case vs. control**	**χ${}_{\text{geno}}^{2}$**	***P*_geno_**	***P*_perm_**	**rs6269 allele count (G/A) Case vs. control**	**χ${}_{\text{alle}}^{2}$**	***P*_alle_**	***P*_perm_**
AA	8/10/8 vs. 11/70/73	13.38	0.001246	0.0015	26/26 vs. 92/216	8.182	0.004232	0.004707
AG	8/38/36 vs. 51/200/209	0.2793	0.8697	0.8506	54/110 vs. 302/618	0.0006405	0.9798	0.9638
GG	3/17/28 vs. 38/175/151	NA	NA	NA	23/73 vs. 251/477	4.229	0.03974	0.03915
ALL	19/65/72 vs. 100/445/433	1.036	0.5956	0.6016	103/209 vs. 645/1311	0.0001699	0.9896	0.974

*NA, not applicable (since there are fewer than 5 observations in some contingency table cells). P_perm_, permutation P-value*.

**Table 6:** Logistic regression analysis of rs6269 in the rs1800497 AA set, the rs1800497 AG set, the rs1800497 GG set and the full set, respectively.

**rs1800497 genotype**	**OR (95% CI)**	**beta**	**Std. Error**	***t*-value**	***P*-value**	***P*_perm_**
AA	2.32 (1.22, 4.41)	0.84061	0.32807	2.562	0.010398	0.00839
AG	1.02 (0.71, 1.48)	0.02271	0.18878	0.120	0.904240	0.9215
GG	0.56 (0.33, 0.95)	−0.58444	0.27256	−2.144	0.03201	0.03067
ALL	1.00 (0.77, 1.31)	0.001047	0.136473	0.008	0.99388	0.9917

*P_perm_, permutation P-value*.

Haplotype-based analysis further supported the interaction between *DRD2*/*ANNK1* and *COMT*. An LD plot of the four *COMT* SNPs is shown in Supplementary Figure 1, indicating that the four SNPs were in one LD block. As estimated by the EM algorithm, there were three major rs6269-rs4633-rs4818-rs4680 haplotypes, G-C-G-G, A-T-C-A and A-C-C-G (their total haplotype frequency [HF] was 97.38% in all the data; see Supplementary Table 8). Detailed analysis denoted that 94.34% of the rs6269 minor allele G was corresponding to haplotype G-C-G-G, 97.75% of rs6269 major allele A was corresponding to haplotype A-T-C-A/A-C-C-G and the *r*^2^ value between rs6269 (minor allele G) and rs6269-rs4633-rs4818-rs4680 haplotypes (reference haplotype G-C-G-G) was 0.95, agreeing with the results of a previous study in a European population (35). Since the rs6269 minor allele G tagged the haplotype rs6269G-rs4633C-rs4818G-rs4680G, we only considered the haplotypes G-C-G-G (reference haplotype) and A-T-C-A/A-C-C-G (alternative haplotype) for the haplotype-based logistic regression analysis. We employed an additive model of the haplotype count of G-C-G-G. As shown in Table 7, the rs6269-rs4633-rs4818-rs4680 haplotype G-C-G-G was associated with an increased PTSD risk (OR = 2.46 and *P* = 0.0144) in the rs1800497 AA set but decreased PTSD risk (OR = 0.535 and *P* = 0.0247) in the rs1800497 GG set. Since rs6269 alleles tagged the rs6269-rs4633-rs4818-rs4680 haplotypes, the results are consistent with results of the subset analysis on the rs6269 allele. Also, the results in the total sample were only replicated with the female sample (details not shown). All the haplotype-based subset analysis results were verified by permutation analysis (Table 7).

**Table 7:** Logistic regression analysis of rs6269-rs4633-rs4818-rs4680 haplotype in the rs1800497 AA set, the rs1800497 AG set, the rs1800497 GG set and the full set, respectively.

**rs1800497 genotype**	**RHF in case**	**RHF in control**	**HF**	**OR**	**STAT**	***P*-value**	***P*_perm_**
AA	0.5238	0.2986	0.327	2.46	5.98	0.0144	0.012
AG	0.3333	0.3222	0.324	1.07	0.127	0.722	0.7081
GG	0.2283	0.3443	0.331	0.535	5.04	0.0247	0.02377
ALL	0.3276	0.3269	0.327	0.997	0.0005	0.982	0.9791

*RHF, reference haplotype (G-C-G-G) frequency. OR, odds ratio for reference haplotype (G-C-G-G) and alternative haplotype (A-T-C-A/A-C-C-G). HF, reference haplotype frequency in all subjects. P_perm_, permutation P-value*.

### Replication of gene–gene interaction

For the replication cohort, only rs1800497 and rs6269 were genotyped. The GF of the two SNPs in cases and controls and results of the HWE test were presented in Supplementary Table 1. We obtained *P*_interaction_ = 0.01329 for the simple logistic regression model of rs1800497 × rs6269; beta = 0.8647 and *P*_interaction_ = 0.0358 for the optimized logistic regression model. Further statistical analysis for subsets could not be carried out since the sample size was too small for each subset, though the same trend was observed. This indicates that the association between PTSD and the *DRD2/ANNK1*–*COMT* gene interaction was preliminarily replicated.

## Discussion

### General discussion

In this study, we investigated gene–gene interaction through studying SNP–SNP interaction. There are 21 statistical tests for screening the 21 pairwise SNP–SNP interactions and multiple testing correction was applied to correct for this. After multiple testing correction, we identified the rs1800497 × rs6269 interaction to be significantly associated with PTSD (*P*_corrected_ < 0.05). Then, all the other tests, including all the subset and haplotype analyses, were performed to explore the properties and details of this interaction; as these were not multiple comparisons of the same subjects, we did not apply multiple testing correction. Consequently, the exact number of statistical tests is 21. The effect size (beta) of the interaction is 0.64 (logarithm of the interaction OR 1.89). To assess power of our study, we utilized Quanto, a tool developed to analyze power and sample size for genetic studies (36) to calculate statistical power, with our study's parameters population risk 0.039, rs1800497 risk allele (A) frequency 0.39, rs6269 risk allele (G) frequency 0.33, interaction OR 1.89, sample size 156(case)/978(control) and significant level (type I error rate) 0.001. The result showed that the power of our study is 57.4%. Since all subjects came from the same community, we assumed that the population stratification in our samples was negligible.

Since PTSD symptoms are highly overlapped with depression symptoms, there is possibility that depression is a confounding factor for our analyses. To assess this issue, we first measured depression with the Center for Epidemiological Studies-Depression (CES-D) Scale (37) in the predominantly adult cohort samples. Then we tried to control for the composition of PTSD symptoms by adding the CES-D scale score as a covariate in our final logistic regression model. The results showed that depression symptom measurement is associated with PTSD (*P* = 4e-23) while it nearly does not affect the association between the gene–gene interaction rs1800497 × rs6269 and PTSD (*P* = 0.00163, very similar to the *P*-value [0.00201] obtained without consideration for depression). This confirmed that the identified association is specific for PTSD.

Our analyses identified that the *DRD2/ANNK1*–*COMT* gene interaction (rs1800497 × rs6269) confers an increased risk of PTSD (*P*_corrected_ = 0.0169155). More specifically, rs1800497 modulates the association directions between the rs6269 allele/rs6269-tagged rs6269-rs4633-rs4818-rs4680 haplotypes and PTSD diagnosis. The associations between PTSD and rs6269/rs6269-rs4633-rs4818-rs4680 haplotypes, in different directions, were observed when rs1800497 AA or GG genotypes were present. These results can only be seen in females based on our data, indicating that gender is an important factor for genetic studies and there might be some potential gene–gene interactions between X-linked genes and dopaminergic genes. Also, taking into account that our results were replicated in Chinese young females who had experienced the earthquake, it seems that at least our results generally apply to female Chinese earthquake survivors. Furthermore, the minor allele frequencies (MAFs) of rs1800497 and rs6269 are 0.39 and 0.33 respectively in our study. According to the 1000 Genomes Phase 3 data (38), the MAFs of the two SNPs in our population are close to those in the East Asian population EAS (MAF = 0.41 and 0.34 respectively) and African population AFR (0.39 and 0.37 respectively), with MAF difference <0.05 for both of the SNPs; they are different from those in Ad Mixed American population AMR (0.31 and 0.31 respectively), European population EUR (0.19 and 0.41 respectively) and South Asian population SAS (0.31 and 0.33 respectively), with MAF difference ≥ 0.05 for at least one of the SNPs. Thus, our results possibly generally apply to East Asian and African.

According to our results, the rs6269 minor allele G-tagged *COMT* rs6269-rs4633-rs4818-rs4680 haplotype G-C-G-G was associated with an increased PTSD risk (OR = 2.46 and *P* = 0.0144) when the rs1800497 genotype AA was present. However, this was associated with a decreased PTSD risk (OR = 0.535 and *P* = 0.0247) when the rs1800497 genotype was GG. In those individuals whose rs1800497 genotype was AG, this haplotype did not show a correlation with PTSD (OR = 1.07, *P* = 0.7081). Previous literature has established that the rs1800497 AA, AG and GG genotypes correspond to low, intermediate and high DRD2 density respectively *in vivo* (39, 40). The rs6269-rs4633-rs4818-rs4680 haplotype affects COMT protein levels and enzyme activity by altering its mRNA secondary structure, producing the following phenotypes: the haplotype G-C-G-G will result in high levels, A-T-C-A will result in normal levels and A-C-C-G will result in low COMT protein levels and enzyme activity (41). Thus, an inference on the biological function and mechanism of the *DRD2*–*COMT* interaction may be as follows. In the case of low DRD2 density, high COMT levels and enzyme activity (resulting in overall low DA signaling) increase the PTSD risk; in the situation of high DRD2 density, the effect of high COMT protein levels and enzyme activity is protective (preventing an overall high DA signaling). By now the abnormalities of the dopaminergic system in PTSD have been uncovered. One recent study found that PTSD subjects have a higher striatal DA transporter density, showing physiological evidence for this hypothesis (42). It has also been reported that, in chronic PTSD subjects, a decline in CNS DA metabolite homovanillic acid concentrations correlates with laboratory-induced symptoms (43). These findings provide a potential explanation for the observed relationship between abnormalities in homeostasis of the dopaminergic system and development of PTSD.

We also performed candidate gene association analyses and gene–environment interaction analyses. We found no statistically significant results, suggesting that these SNPs confer a higher PTSD risk only by gene–gene interactions, based on our cohort. The SNP rs1800497, also known as *DRD2* TaqIA, has been investigated in many studies. This SNP has been considered a marker for altered DRD2 density. It has also been argued that it might be a marker for some functional variant of *DRD2* or be indirectly involved in *DRD2* expression (24). When we split our complete data by the rs6269 genotypes GG, GA and AA, rs1800497 was only associated with PTSD (*P* < 0.05) in the rs6269 GG set, which had a relatively small sample size (19 cases and 100 controls). Thus, it is more likely that rs1800497 controls the association directions of rs6269 than the opposite situation. Goenjian et al. (44) examined the effect of rs6269 on PTSD severity, and no significant relationships were found. The findings of our study provide a possible explanation for this negative result and show the advantage of including gene–gene interactions in explaining the genetic basis of PTSD. The *DRD2*–*COMT* interaction has been reported to be associated with working memory (45). As deficits in working memory have been highlighted in PTSD patients (46), whether the association between the *DRD2*–*COMT* interaction and PTSD is mediated by affecting the working memory deserves further research. In our study, we found the *DRD2*–*COMT* interaction signal to be present in women only. Possible explanations include the influence of sex hormones on dopaminergic neurotransmission or the effect of having many more women than men enrolled in the study and hence insufficient power to show an effect in men.

As described above, our findings are quite illuminating with respect to the existing literature. This indicates that studying gene–gene interaction has great potential for future investigation of the association between genes and mental disorders, especially when seemingly significant alterations in relevant genes demonstrate no discernable association to disease manifestation. Furthermore, this also shows that genes may contribute to mental disorders in a nonlinear fashion.

### Limitations

There are some obvious limitations in our study. First, the overall sample size is modest compared other genetic studies and thus negative findings should be interpreted with caution due to the power issues. Our study examined effects of only one trauma exposure, the 2008 earthquake. Thus, the results would need to be investigated in other PTSD study cohorts exposed to different types of trauma. Another limitation is that *DSM-5* PTSD diagnosis was inferred according to PCL-5, which is a self-report measure. Therefore, further studies using clinician-administered instruments are warranted. Since the replication cohort only contains young females, it is preliminary and limited. The last limitation is that we only explored three dopaminergic neurotransmission-related loci and seven SNPs of the three loci. In the future, more dopamine-relevant loci and their genetic variants should be tested to detect gene–gene interaction effects on PTSD more completely.

## Conclusions

Despite these limitations, we first found that the *DRD2*/*ANNK1*–*COMT* interaction confers a higher PTSD risk. Since the SNPs correspond to polymorphisms of protein levels and activity, the association was inferred to be at a protein–protein interaction and DA homeostasis level, providing a potential origin of DA homeostasis abnormalities in PTSD development. Through gene–gene interaction analysis, our study has provided new insight into the biological mechanisms and genetic architectures of PTSD. Furthermore, our study suggests that taking gene–gene interaction into account is an important method for evaluating the potential genetic contribution to psychopathology rather than considering only single genes or polygenic risk scores. For precision medicine, the genotype combinations of SNPs rs1800497 and rs6269 will assistant early prevention.

## Author contributions

LW and KZ conceived and designed the study. LW was in charge of the sample collection. KZ performed the statistical analysis. CC managed the genotyping. PL and SL contributed to the sample collection. GL, XZ, and RF helped to revise the manuscript. KZ and LW wrote the manuscript. IL revised the manuscript. All authors read and approved the final version of the manuscript.

### Conflict of interest statement

The authors declare that the research was conducted in the absence of any commercial or financial relationships that could be construed as a potential conflict of interest.

## References

[1] American Psychiatric Association Diagnostic and Statistical Manual of Mental Disorders, 5th Edn. Washington, DC: American Psychiatric Associaton (2013).

[2] Koenen KC Ratanatharathorn A Ng L McLaughlin KA Bromet EJ Stein DJ. Posttraumatic stress disorder in the World Mental Health Surveys. Psychol Med. (2017) 47:2260–74. 10.1017/S0033291717000708 28385165 PMC6034513

[3] Broekman BF Olff M Boer F. The genetic background to PTSD. Neurosci Biobehav Rev. (2007) 31:348–62. 10.1016/j.neubiorev.2006.10.001 17126903

[4] Afifi TO Asmundson GJ Taylor S Jang KL. The role of genes and environment on trauma exposure and posttraumatic stress disorder symptoms: a review of twin studies. Clin Psychol Rev. (2010) 30:101–12. 10.1016/j.cpr.2009.10.002 19892451

[5] Olff M Langeland W Gersons BP. The psychobiology of PTSD: coping with trauma. Psychoneuroendocrinology (2005) 30:974–82. 10.1016/j.psyneuen.2005.04.009 15964146

[6] Duncan LE Ratanatharathorn A Aiello AE Almli LM Amstadter AB Ashley-Koch AE. Largest GWAS of PTSD (N = 20 070) yields genetic overlap with schizophrenia and sex differences in heritability. Mol Psychiatry (2017) 23:666–73. 10.1038/mp.2017.77 28439101 PMC5696105

[7] Zhang K Qu S Chang S Li G Cao C Fang K. An overview of posttraumatic stress disorder genetic studies by analyzing and integrating genetic data into genetic database PTSDgene. Neurosci Biobehav Rev. (2017) 83:647–56. 10.1016/j.neubiorev.2017.08.021 28888533

[8] Smoller JW. The genetics of stress-related disorders: PTSD, depression, and anxiety disorders. Neuropsychopharmacology (2016) 41:297–319. 10.1038/npp.2015.266 26321314 PMC4677147

[9] Banerjee SB Morrison FG Ressler KJ. Genetic approaches for the study of PTSD: advances and challenges. Neurosci Lett. (2017) 649:139–46. 10.1016/j.neulet.2017.02.058 28242325 PMC5470933

[10] Schizophrenia Working Group of the Psychiatric Genomics Consortium. Biological insights from 108 schizophrenia-associated genetic loci. Nature (2014) 511:421–7. 10.1038/nature13595 25056061 PMC4112379

[11] Hyde CL Nagle MW Tian C Chen X Paciga SA Wendland JR. Identification of 15 genetic loci associated with risk of major depression in individuals of European descent. Nat Genet. (2016) 48:1031–6. 10.1038/ng.3623 27479909 PMC5706769

[12] Chen DT Jiang X Akula N Shugart YY Wendland JR Steele CJ. Genome-wide association study meta-analysis of European and Asian-ancestry samples identifies three novel loci associated with bipolar disorder. Mol Psychiatry (2013) 18:195–205. 10.1038/mp.2011.157 22182935

[13] Collins FS Varmus H. A new initiative on precision medicine. N Engl J Med. (2015) 372:793–5. 10.1056/NEJMp1500523 25635347 PMC5101938

[14] Phillips PC. Epistasis–the essential role of gene interactions in the structure and evolution of genetic systems. Nat Rev Genet. (2008) 9:855–67. 10.1038/nrg2452 18852697 PMC2689140

[15] Cordell HJ. Detecting gene-gene interactions that underlie human diseases. Nat Rev Genet. (2009) 10:392–404. 10.1038/nrg2579 19434077 PMC2872761

[16] Wei WH Hemani G Haley CS. Detecting epistasis in human complex traits. Nat Rev Genet. (2014) 15:722–33. 10.1038/nrg3747 25200660

[17] Frazer KA Murray SS Schork NJ Topol EJ. Human genetic variation and its contribution to complex traits. Nat Rev Genet. (2009) 10:241–51. 10.1038/nrg2554 19293820

[18] Girault JA Greengard P. The neurobiology of dopamine signaling. Arch Neurol. (2004) 61:641–4. 10.1001/archneur.61.5.641 15148138

[19] Cabib S Puglisi-Allegra S. The mesoaccumbens dopamine in coping with stress. Neurosci Biobehav Rev. (2012) 36:79–89. 10.1016/j.neubiorev.2011.04.012 21565217

[20] Hu H. Reward and Aversion. Annu Rev Neurosci. (2016) 39:297–324. 10.1146/annurev-neuro-070815-014106 27145915

[21] Kienast T Heinz A. Dopamine and the diseased brain. CNS Neurol Disord Drug Targets (2006) 5:109-31. 10.2174/187152706784111560 16613557

[22] Faraone SV Khan SA. Candidate gene studies of attention-deficit/hyperactivity disorder. J Clin Psychiatry (2006) 67 (Suppl. 8):13–20. 16961425

[23] Bandelow B Baldwin D Abelli M Bolea-Alamanac B Bourin M Chamberlain SR. Biological markers for anxiety disorders, OCD and PTSD: a consensus statement. Part II: neurochemistry, neurophysiology and neurocognition. World J Biol Psychiatry (2017) 18:162–214. 10.1080/15622975.2016.1190867 27419272 PMC5341771

[24] Nemoda Z Szekely A Sasvari-Szekely M. Psychopathological aspects of dopaminergic gene polymorphisms in adolescence and young adulthood. Neurosci Biobehav Rev. (2011) 35:1665–86. 10.1016/j.neubiorev.2011.04.002 21527290 PMC3133854

[25] Li L Bao Y He S Wang G Guan Y Ma D. The association between genetic variants in the dopaminergic system and posttraumatic stress disorder: a meta-analysis. Medicine (Baltimore) (2016) 95:e3074. 10.1097/MD.0000000000003074 26986136 PMC4839917

[26] Cao C Wang L Cao X Dong C Liu P Luo S. Support for the association between RORA gene polymorphisms and the DSM-5 posttraumatic stress disorder symptoms in male earthquake survivors in China. Asian J Psychiatry (2017) 25:138–41. 10.1016/j.ajp.2016.10.028 28262136

[27] Liu P Wang L Cao C Wang R Zhang J Zhang B. The underlying dimensions of DSM-5 posttraumatic stress disorder symptoms in an epidemiological sample of Chinese earthquake survivors. J Anxiety Disord (2014) 28:345–51. 10.1016/j.janxdis.2014.03.008 24792723

[28] Blevins CA Weathers FW Davis MT Witte TK Domino JL. The posttraumatic stress disorder checklist for DSM-5 (PCL-5): development and initial psychometric evaluation. J Trauma Stress (2015) 28:489–98. 10.1002/jts.22059 26606250

[29] Wortmann JH Jordan AH Weathers FW Resick PA Dondanville KA Hall-Clark B. Psychometric analysis of the PTSD Checklist-5 (PCL-5) among treatment-seeking military service members. Psychol Assess (2016) 28:1392–403. 10.1037/pas0000260 26751087

[30] Wigginton JE Cutler DJ Abecasis GR. A note on exact tests of Hardy-Weinberg equilibrium. Am J Hum Genet. (2005) 76:887–93. 10.1086/429864 15789306 PMC1199378

[31] Purcell S Neale B Todd-Brown K Thomas L Ferreira MA Bender D. PLINK: a tool set for whole-genome association and population-based linkage analyses. Am J Hum Genet (2007) 81:559–75. 10.1086/519795 17701901 PMC1950838

[32] Barrett JC. Haploview: visualization and analysis of SNP genotype data. Cold Spring Harb Protoc. (2009) 2009:pdb ip71. 10.1101/pdb.ip71 20147036

[33] Sakamoto Y Ishiguro M Kitagawa G Akaike Information Criterion Statistics: Boston, MA: D. Reidel Publishing Company (1986).

[34] Hastie TJ Pregibon D Statistical Models in S. In: Chambers JM Hastie TJ editors. Generalized linear models. Pacific Grove, CA: Wadsworth & Brooks/Cole (1992). p. 195–248.

[35] Roten LT Fenstad MH Forsmo S Johnson MP Moses EK Austgulen R. A low COMT activity haplotype is associated with recurrent preeclampsia in a Norwegian population cohort (HUNT2). Mol Hum Reprod. (2011) 17:439–46. 10.1093/molehr/gar014 21355050 PMC3116680

[36] Gauderman WJ. Sample size requirements for association studies of gene-gene interaction. Am J Epidemiol. (2002) 155:478–84. 10.1093/aje/155.5.478 11867360

[37] Radloff LS The CES-D scale: a self-report depression scale for research in the general population. Appl. Psychol. Meas. (1977) 1:385–401. 10.1177/014662167700100306

[38] Genomes Project C Auton A Brooks LD Durbin RM Garrison EP Kang HM. A global reference for human genetic variation. Nature (2015) 526:68–74. 10.1038/nature15393 26432245 PMC4750478

[39] Jonsson EG Nothen MM Grunhage F Farde L Nakashima Y Propping P. Polymorphisms in the dopamine D2 receptor gene and their relationships to striatal dopamine receptor density of healthy volunteers. Mol Psychiatry (1999) 4:290–6. 10395223 10.1038/sj.mp.4000532

[40] Noble EP. D2 dopamine receptor gene in psychiatric and neurologic disorders and its phenotypes. Am J Med Genet B Neuropsychiatr Genet (2003) 116B:103–25. 10.1002/ajmg.b.10005 12497624

[41] Nackley AG Shabalina SA Tchivileva IE Satterfield K Korchynskyi O Makarov SS. Human catechol-O-methyltransferase haplotypes modulate protein expression by altering mRNA secondary structure. Science (2006) 314:1930–3. 10.1126/science.1131262 17185601

[42] Hoexter MQ Fadel G Felicio AC Calzavara MB Batista IR Reis MA. Higher striatal dopamine transporter density in PTSD: an *in vivo* SPECT study with [(99m)Tc]TRODAT-1. Psychopharmacology (Berl) (2012) 224:337–45. 10.1007/s00213-012-2755-4 22700036

[43] Geracioti TD Jr Jefferson-Wilson L Strawn JR Baker DG Dashevsky BA Horn PS. Effect of traumatic imagery on cerebrospinal fluid dopamine and serotonin metabolites in posttraumatic stress disorder. J Psychiatr Res. (2013) 47:995–8. 10.1016/j.jpsychires.2013.01.023 23540599

[44] Goenjian AK Noble EP Steinberg AM Walling DP Stepanyan ST Dandekar S. Association of COMT and TPH-2 genes with DSM-5 based PTSD symptoms. J Affect Disord. (2015) 172:472–8. 10.1016/j.jad.2014.10.034 25451452

[45] Stelzel C Basten U Montag C Reuter M Fiebach CJ. Effects of dopamine-related gene-gene interactions on working memory component processes. Eur J Neurosci. (2009) 29:1056–63. 10.1111/j.1460-9568.2009.06647.x 19291230

[46] Scott JC Matt GE Wrocklage KM Crnich C Jordan J Southwick SM. A quantitative meta-analysis of neurocognitive functioning in posttraumatic stress disorder. Psychol Bull. (2015) 141:105–40. 10.1037/a0038039 25365762 PMC4293317

