# Supplementary material for: A DRD2/ANNK1–COMT Interaction, Consisting of Functional Variants, Confers Risk of Post-traumatic Stress Disorder in Traumatized Chinese
Source: Front Psychiatry. 2018 Apr 30;9:170. doi: 10.3389/fpsyt.2018.00170 (PMC5936991; doi:10.3389/fpsyt.2018.00170)
Supplement: Supplementary file 1 [file Data_Sheet_1.doc]

**Supplementary materials**

**A *DRD2/ANNK1*–*COMT* interaction, consisting of functional variants, confers risk of post-traumatic stress disorder in traumatized Chinese**

Kunlin Zhang ^1,2^, Li Wang ^1,2^,*, Chengqi Cao ^1,3^, Gen Li ^1,2^, Ruojiao Fang ^1,2^, Ping Liu^4^, Shu Luo^4^, Xiangyang Zhang ^1,2^, Israel Liberzon ^5, 6^

^1^ Laboratory for Traumatic Stress studies and Center for Genetics and BioMedical Informatics Research, CAS Key Laboratory of Mental Health, Institute of Psychology, Beijing 100101, China

^2^ Department of Psychology, University of Chinese Academy of Sciences, Beijing 100049, China

^3^ Shenzhen Key Laboratory of Affective and Social Cognitive Science, Shenzhen University, Shenzhen 518060, China

^4^ People’s Hospital of Deyang City, Deyang, Sichuan 618000, China

^5^ Department of Psychiatry, University of Michigan and Mental Health Service, VA Ann Arbor Healthcare System, Ann Arbor, MI 48109-2700, USA

^6^ Department of Psychology, University of Michigan, Ann Arbor, MI 48109-2700, USA

* **Corresponding author:**

Li Wang, Institute of Psychology, Chinese Academy of Sciences, 16 Lincui Road, Beijing 100101, China.

E-mail address: wangli1@psych.ac.cn

**Running title:** *DRD2*/*ANNK1*–*COMT* interaction confers PTSD risk

**Supplementary Table 1**. Detailed information of the seven SNPs of the three dopaminergic genes.

| **SNP** | **Chr.** | **Position** ^a^ | **Allele ^b^** | **Gene** | **Possible function** | **GF**  **in cases** ^c^ | **GF**  **in controls ^d^** | **HWE test**  ***P* value**  **(case/control) ^e^** |
| --- | --- | --- | --- | --- | --- | --- | --- | --- |
| rs1800497 | 11 | 113270828 | A/G | *DRD2*/*ANNK* | Missense (E->K) for *ANNK*. Affect DRD2 density (Noble et al., *Am J Med Genet B Neuropsychiatr Genet.* 2003). | AA/AG/GG  26/82/48  5/13/14 (replicaton cohort) | AA/AG/GG  154/460/364  87/262/227 (replication cohort) | 0.4164/0.6875  0.4693/0.4262 (replication cohort) |
|  |  |  |  |  |  |  |  |  |
| rs1801028 | 11 | 113283484 | C/G | *DRD2* | Missense (S->C) for *DRD2*. | CC/CG/GG  1/10/145 | CC/CG/GG  1/70/907 | 0.1972/1 |
|  |  |  |  |  |  |  |  |  |
| rs6269 | 22 | 19949952 | G/A | *COMT* | Affect COMT level and enzyme activity (rs6269-rs4633-rs4818-rs4680 haplotypes) (Nackley et al., *Science* 2006). | GG/GA/AA  19/65/72  3/14/15 (replicaton cohort) | GG/GA/AA  100/445/433  60/252/268 (replicaton cohort) | 0.4715/0.3856  1/0.9244 (replication cohort) |
|  |  |  |  |  |  |  |  |  |
| rs4633 | 22 | 19950235 | T/C | *COMT* | Synonymous (H->H) | TT/TC/CC  11/55/90 | TT/TC/CC  68/378/532 | 0.5201/0.9343 |
|  |  |  |  |  |  |  |  |  |
| rs4818 | 22 | 19951207 | G/C | *COMT* | Synonymous (L->L) | GG/GC/CC  17/65/74 | GG/GC/CC  94/445/439 | 0.7112/0.2425 |
|  |  |  |  |  |  |  |  |  |
| rs4680 | 22 | 19951271 | A/G | *COMT* | Missense (V->M) | AA/AG/GG  9/60/87 | AA/AG/GG  68/391/519 | 0.8338/0.6847 |
|  |  |  |  |  |  |  |  |  |
| rs1611115 | 9 | 136500515 | T/C | *DBH* | C-1021T (nearGene-5). Associated with plasma DBH levels (Garland et al., *Am J Physiol Heart Circ Physiol.* 2007). | TT/TC/CC  7/41/108 | TT/TC/CC  27/287/664 | 0.2657/0.6564 |

Chr., chromosome. GF, genotype frequency. HWE, Hardy-Weinberg Equilibrium.

^a^ based on hg19.

^b^ minor/major.

^c, d, e^ all refer to the discovery cohort unless “replication cohort” is mentioned.

**Supplementary Table 2**. Results of single SNP-based analysis for the seven dopaminergic gene SNPs.

| **SNP** | **Allele ^a^** | **MAF**  **in cases** | **MAF**  **in controls** | **G**×**E ^b^** | **OR (95% CI)** | **Std. Error** | ***t* value** | ***P* value** |
| --- | --- | --- | --- | --- | --- | --- | --- | --- |
| rs1800497 | A/G | 0.4295 | 0.3926 | N | 1.19 (0.93, 1.53) | 0.1276 | 1.382 | 0.167 |
|  |  |  |  | Y | 1.18 (0.67, 2.07) | 0.2863 | 0.5763 | 0.5644 |
|  |  |  |  | inter | 1.00 (0.88, 1.14) | 0.06601 | 0.04414 | 0.9648 |
| rs1801028 | C/G | 0.0385 | 0.0368 | N | 1.06 (0.56, 2.01) | 0.3277 | 0.1657 | 0.8684 |
|  |  |  |  | Y | 1.32 (0.34, 5.09) | 0.6903 | 0.3981 | 0.6905 |
|  |  |  |  | inter | 0.94 (0.66, 1.33) | 0.1767 | -0.3558 | 0.722 |
| rs6269 | G/A | 0.3301 | 0.3298 | N | 1.00 (0.77, 1.31) | 0.1365 | 0.007674 | 0.9939 |
|  |  |  |  | Y | 1.35 (0.73, 2.50) | 0.3148 | 0.9551 | 0.3395 |
|  |  |  |  | inter | 0.92 (0.80, 1.07) | 0.07538 | -1.05 | 0.2937 |
| rs4633 | T/C | 0.2468 | 0.2628 | N | 0.86 (0.65, 1.15) | 0.1451 | -1.004 | 0.3153 |
|  |  |  |  | Y | 0.73 (0.37, 1.42) | 0.3416 | -0.9268 | 0.354 |
|  |  |  |  | inter | 1.05 (0.89, 1.22) | 0.07965 | 0.5562 | 0.5781 |
| rs4818 | G/C | 0.3173 | 0.3236 | N | 0.96 (0.73, 1.26) | 0.1383 | -0.2775 | 0.7814 |
|  |  |  |  | Y | 1.34 (0.72, 2.49) | 0.316 | 0.9308 | 0.352 |
|  |  |  |  | inter | 0.92 (0.79, 1.06) | 0.07546 | -1.161 | 0.2455 |
| rs4680 | A/G | 0.2500 | 0.2694 | N | 0.84 (0.63, 1.12) | 0.1467 | -1.206 | 0.2276 |
|  |  |  |  | Y | 0.91 (0.47, 1.77) | 0.3408 | -0.278 | 0.781 |
|  |  |  |  | inter | 0.98 (0.84, 1.15) | 0.0805 | -0.2666 | 0.7898 |
| rs1611115 | T/C | 0.1763 | 0.1743 | N | 1.04 (0.75, 1.43) | 0.1654 | 0.2174 | 0.8279 |
|  |  |  |  | Y | 0.83 (0.40, 1.72) | 0.3714 | -0.5076 | 0.6118 |
|  |  |  |  | inter | 1.06 (0.89, 1.26) | 0.08726 | 0.6834 | 0.4944 |

MAF, minor allele frequency. G×E, gene–environment interaction (SNP×trauma exposure).

^a^ minor/major.

^b^ N: main effect of SNP based on logistic regression without G×E; Y: main effect of SNP based on logistic regression with G×E; inter: results for G×E based on logistic regression with G×E.

**Supplementary Table 3**. Gene–gene interaction screening results for all the 21 SNP pairs.

| **CHR1** | **SNP1** | **CHR2** | **SNP2** | **OR_INT** | **STAT** | ***P*_interaction_** | ***P*_corrected_** |
| --- | --- | --- | --- | --- | --- | --- | --- |
| 9 | rs1611115 | 11 | rs1800497 | 1.37891 | 1.79678 | 0.1801 | 1 |
| 9 | rs1611115 | 11 | rs1801028 | 0.809775 | 0.0918206 | 0.7619 | 1 |
| 9 | rs1611115 | 22 | rs4633 | 1.06011 | 0.0486277 | 0.8255 | 1 |
| 9 | rs1611115 | 22 | rs4680 | 0.980111 | 0.00545355 | 0.9411 | 1 |
| 9 | rs1611115 | 22 | rs4818 | 0.83866 | 0.475501 | 0.4905 | 1 |
| 9 | rs1611115 | 22 | rs6269 | 0.795824 | 0.818273 | 0.3657 | 1 |
| 11 | rs1800497 | 11 | rs1801028 | 0.794753 | 0.101158 | 0.7504 | 1 |
| 11 | rs1800497 | 22 | rs4633 | 0.939989 | 0.0924582 | 0.7611 | 1 |
| 11 | rs1800497 | 22 | rs4680 | 0.835469 | 0.760815 | 0.3831 | 1 |
| 11 | rs1800497 | 22 | rs4818 | 1.88327 | 10.8138 | 0.001008 | 0.021168 |
| 11 | rs1800497 | 22 | rs6269 | 1.89024 | 11.2287 | 0.0008055 | 0.0169155 |
| 11 | rs1801028 | 22 | rs4633 | 1.51558 | 0.70196 | 0.4021 | 1 |
| 11 | rs1801028 | 22 | rs4680 | 1.69741 | 1.1732 | 0.2787 | 1 |
| 11 | rs1801028 | 22 | rs4818 | 0.297673 | 3.40554 | 0.06498 | 1 |
| 11 | rs1801028 | 22 | rs6269 | 0.366973 | 2.87558 | 0.08993 | 1 |
| 22 | rs4633 | 22 | rs4680 | 0.93523 | 0.0844813 | 0.7713 | 1 |
| 22 | rs4633 | 22 | rs4818 | 0.775442 | 0.793223 | 0.3731 | 1 |
| 22 | rs4633 | 22 | rs6269 | 0.74624 | 1.05219 | 0.305 | 1 |
| 22 | rs4680 | 22 | rs4818 | 0.945957 | 0.0406266 | 0.8403 | 1 |
| 22 | rs4680 | 22 | rs6269 | 1.0235 | 0.00810058 | 0.9283 | 1 |
| 22 | rs4818 | 22 | rs6269 | 1.23301 | 1.12864 | 0.2881 | 1 |

**Supplementary Table 4**. Summary of the initial logistic regression model of rs1800497×rs6269.

| **Variable** | **beta** | **Std. Error** | ***t* value** | ***P* value** |
| --- | --- | --- | --- | --- |
| rs1800497×rs6269 | 0.934398 | 0.463317 | 2.017 | 0.04372 |
| rs1800497 | -0.514510 | 0.437936 | -1.175 | 0.24005 |
| rs6269 | -0.527566 | 0.530608 | -0.994 | 0.32009 |
| Gender | 0.537655 | 0.202961 | 2.649 | 0.00807 |
| Age | 0.072154 | 0.010778 | 6.694 | 2.17e-11 |
| Trauma exposure | 0.251135 | 0.104828 | 2.396 | 0.01659 |
| rs1800497×trauma exposure | 0.071589 | 0.100320 | 0.714 | 0.47547 |
| rs6269×trauma exposure | -0.003467 | 0.125750 | -0.028 | 0.97801 |
| rs1800497×rs6269×trauma exposure | -0.086188 | 0.109511 | -0.787 | 0.43127 |

**Supplementary Table 5**. Demography of the rs1800497 AA set, the rs1800497 AG set, the rs1800497 GG set and the full set, respectively.

| **rs1800497 genotype** | **No. of samples**  **(female/male)** | **Age of samples**  **(mean±sd)** | **No. of cases**  **(female/male)** | **Age of cases**  **(mean±sd)** | **No. of controls**  **(female/male)** | **Age of controls**  **(mean±sd)** |
| --- | --- | --- | --- | --- | --- | --- |
| AA | 180 (123/57) | 47.63***±***10.93 | 26 (17/9) | 52.46***±***10.49 | 154 (106/48) | 46.82***±***10.83 |
| AG | 542 (369/173) | 47.87***±***9.90 | 82 (58/24) | 52.33***±***8.44 | 460 (311/149) | 47.07***±***9.94 |
| GG | 412 (281/131) | 48.55***±***9.59 | 48 (40/8) | 52.90***±***7.88 | 364 (241/123) | 47.97***±***9.65 |
| ALL | 1134 (773/361) | 48.08***±***9.96 | 156 (115/41) | 52.53***±***8.59 | 978 (658/320) | 47.37***±***9.98 |

**Supplementary Table 6**. Logistic regression analysis of rs6269 for females in the rs1800497 AA set, the rs1800497 AG set, the rs1800497 GG set and the full set, respectively.

| **rs1800497 genotype** | **OR (95% CI)** | **beta** | **Std. Error** | ***t* value** | ***P* value** | ***P*_perm_** |
| --- | --- | --- | --- | --- | --- | --- |
| AA | 2.64 (1.17, 5.96) | 0.9704 | 0.416 | 2.333 | 0.01967 | 0.01617 |
| AG | 1.24 (0.79, 1.93) | 0.2119 | 0.2279 | 0.9303 | 0.3522 | 0.3352 |
| GG | 0.52 (0.29, 0.94) | -0.6566 | 0.3051 | -2.152 | 0.03138 | 0.03313 |
| ALL | 1.05 (0.76, 1.44) | 0.04593 | 0.1619 | 0.2821 | 0.7778 | 0.8035 |

*P*_perm_, permutation *P* value.

**Supplementary Table 7**. Logistic regression analysis of rs6269 for males in the rs1800497 AA set, the rs1800497 AG set, the rs1800497 GG set and the full set, respectively.

| **rs1800497 genotype** | **OR (95% CI)** | **beta** | **Std. Error** | ***t* value** | ***P* value** | ***P*_perm_** |
| --- | --- | --- | --- | --- | --- | --- |
| AA | 1.95 (0.66, 5.75) | 0.6699 | 0.5507 | 1.216 | 0.2239 | 0.2337 |
| AG | 0.67 (0.33, 1.34) | -0.4014 | 0.3547 | -1.132 | 0.2577 | 0.26 |
| GG | 0.78 (0.23, 2.63) | -0.2476 | 0.6203 | -0.3991 | 0.6898 | 0.6962 |
| ALL | 0.86 (0.51, 1.43) | -0.1558 | 0.2605 | -0.5983 | 0.5496 | 0.553 |

*P*_perm_, permutation *P* value.

**Supplementary Table 8**. Haplotype (rs6269-rs4633-rs4818-rs4680) frequencies in the rs1800497 AA set, the rs1800497 AG set, the rs1800497 GG set and the full set, respectively.

| **Haplotype** | **HF in**  **rs1800497 AA set** | **HF in**  **rs1800497 AG set** | **HF in**  **rs1800497 GG set** | **HF in**  **rs1800497 full set** |
| --- | --- | --- | --- | --- |
| G-C-G-G | 0.3165 | 0.3173 | 0.3228 | 0.3187 |
| A-T-C-A | 0.2387 | 0.2508 | 0.2597 | 0.2521 |
| A-C-C-G | 0.4026 | 0.4059 | 0.3980 | 0.4030 |
| others | 0.0422 | 0.0260 | 0.0195 | 0.0262 |
| Total | 1 | 1 | 1 | 1 |

HF, haplotype frequency.

**
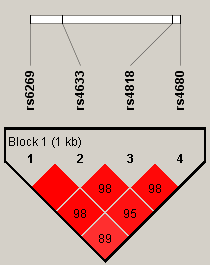

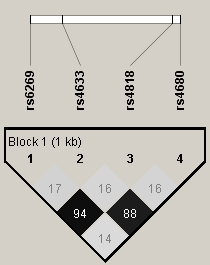
**

**Supplementary Figure 1**. Linkage disequilibrium (LD) plot of four *COMT* SNPs (rs6269, rs4633, rs4818 and rs4680). The left is based on the LD measurement *D’* and the right is based on the LD measurement *r^2^*.
